# Molecular detection of *Toxoplasma gondii* in marine fish from Tunisia: First report in the Southern Mediterranean Sea

**DOI:** 10.1007/s00436-026-08714-1

**Published:** 2026-06-23

**Authors:** Ghozzi Khemissa, Ibtissem Lahmar, Anna Maria Fausta Marino, Alessandra Aparo, Rafika Challouf, Rym Ben Dhiab, Raja Chaâbane Banaoues, Najoua Haouas

**Affiliations:** 1 National Institute of Marine Sciences and Technologies, Marine Biodiversity Laboratory - LR16INSTM02, University of Carthage, 28 Street of March 02, 1934, Salammbô, 2025 Tunisia; 2 Faculty of Pharmacy, Laboratory of Medical and Molecular Parasitology-Mycology LP3M (code LR12ES08), Department of Clinical Biology B, Monastir University, Monastir, 5000 Tunisia; 3 National Reference Centre for Toxoplasmosis, Istituto Zooprofilattico Sperimentale della Sicilia, Catania, Italy

**Keywords:** *T. gondii*, Fish, Habitat, Feeding habit, transmission

## Abstract

Toxoplasmosis, caused by *Toxoplasma gondii* (*T. gondii*), is a ubiquitous zoonotic parasitic disease increasingly reported in marine ecosystems, raising concerns about seafood contamination and potential human exposure. Consumption of raw or undercooked fish may therefore represent a potential risk to consumers. This study aimed to investigate the presence of *T. gondii* DNA in marine fish from the Tunisian coast and to assess their potential role as indicators of environmental contamination. A total of 559 specimens, representing 32 species, were collected and grouped into 100 pooled samples by species. Tissue samples (gills, skin/muscle, and intestine) were analyzed using real-time PCR. Overall, 16% (16/100) of pooled samples were tested positive for *T. gondii*, involving 13 fish species. Positive detections were observed in both wild and farmed fish, including caged *Sparus aurata*. Gill tissues showed the highest frequency of detection (9/16), followed by lower detection rates in skin/muscle (4/16) and intestinal (3/16) samples. Demersal species showed the highest number of positive pools, followed by pelagic and benthopelagic specimens. However, Carnivorous fish showed higher detection rates (36%) compared to omnivorous (31.6%) and herbivorous species (14.3%). This survey provides the first molecular evidence of *T. gondii* in marine fish from Tunisia and highlights the potential role of certain species as indicators of environmental contamination and passive carriers of this parasite within marine ecosystems.

## Introduction

The apicomplexan *Toxoplasma. gondii* is an obligate intracellular parasite capable of infecting nearly all warm-blooded animals, including humans (Dubey 2004). Its life cycle involves domestic and wild felids as definitive hosts and a wide range of mammals and birds, as intermediate hosts. Oocysts shed in felid feces are highly resistant and widely dispersed in the environment, making toxoplasmosis a major water- and food-borne zoonotic disease worldwide (Karanis et al. 2013).

Toxoplasmosis is endemic in Tunisia, where a seroprevalence of 44.4% has been reported among blood donors, mainly reflecting past infection (IgG), with no evidence of recent infection based on IgG avidity testing (Lachkhem et al. 2020). The parasite is also widespread in domestic and wild animals, contributing to significant foodborne and environmental risks. Seroprevalence ranges from 19.7 to 40.2% in sheep, 30–34.5% in goats, and 12–19% in cattle (Lahmar et al. 2015; Amairia et al. 2016; Rouatbi et al. 2016; Guessmi et al. 2023). High seroprevalence levels have also been reported in poultry, reaching up to 100% in free-range chickens (Boughattas and Bouratbine 2014; Lachkhem et al. 2021). In addition, the molecular prevalence of *T. gondii* in industrial poultry meat from Tunisia was estimated at 20% (Zrelli et al.^2022^. 2021). In slaughtered equids from northern Tunisia, overall *T. gondii* molecular prevalence was about 39.7%, with a particularly high rate in donkeys (66.2%), while horses and mules showed lower prevalences (26.1% and 18.7%, respectively) (Amairia et al. 2023). Serological prevalence in dromedary camels was estimated at 29.8% (Jeljli et al. 2024), while wild desert rodents showed high molecular and serological prevalences (39% and 34.9%, respectively), with some species exhibiting even greater infection rates **(**Bouaicha et al. 2025). Collectively, these findings indicate extensive environmental contamination and a sustained risk of transmission to humans. Oocysts released into the environment can be transported via runoff and drainage systems, leading to contamination of freshwater and marine ecosystems. In marine environments, their dispersal is influenced by complex interactions involving suspended particles, biofilms, gastropods, and other invertebrates. Extracellular polymeric substances (EPS) may further facilitate oocyst aggregation and attachment to marine surfaces such as biofilms and seaweeds (Shapiro et al. 2009; Nayeri et al. 2021).

Over recent decades, *T. gondii* has been increasingly detected in marine organisms worldwide, including cetaceans, pinnipeds, sirenians, and sea otters, with reported clinical disease and population-level impacts in some species (Cole et al. 2000; Dubey et al. 2003; Van de Velde et al. 2016; Li et al. 2022). High prevalence has been reported globally, particularly in sea otters (54.8%) and cetaceans (30.9%) (Ahmadpour et al. 2022). Filter-feeding bivalves such as mussels, oysters, and clams can concentrate oocysts and act as important paratenic hosts in marine transmission pathways (Marangi et al. 2015; Tedde et al. 2019; Cong et al. 2017; Ghozzi et al. 2017).

Recent evidence also suggests that fish may act as mechanical carriers of *T. gondii* oocysts, facilitating their dissemination in marine ecosystems (Massie et al. 2010; Marino et al. 2019). Experimental studies have demonstrated that filter-feeding fish can retain oocysts from seawater, potentially contributing to environmental spread. Although fish are not suitable hosts for parasite replication under most natural conditions, detection of *T. gondii* DNA in wild fish has been reported in Asia and the Mediterranean basin (Zhang et al. 2014; Marino et al. 2019). Laboratory studies further indicate that parasite invasion and replication are temperature-dependent, occurring above 27–30 °C (Yang et al. 2020), raising concerns that rising sea surface temperatures due to climate change may increase environmental suitability for parasite survival and transmission.

In Tunisia and across the Mediterranean, fish and seafood represent a major dietary component and an important economic resource, with annual fish consumption estimated at 15 kg per capita and a production exceeding 137,000 tons (APIA 2023). Given the increasing recognition of protozoan parasites in marine food webs, the potential public health risk associated with consumption of contaminated seafood warrants attention. However, to our knowledge, no updated information is available on the occurrence of *T. gondii* in Tunisian marine fish.

Therefore, this study aimed to investigate the presence of *T. gondii* in marine fish from the Tunisian coast, to assess its tissue distribution, and to evaluate the potential role of fish as environmental indicators and passive vectors in marine ecosystems.

## Materials and methods

### Fish sampling and dissection

Fish sampling was carried out between February 2020 and July 2022. A total of 559 individual fish, representing 32 species, were collected from Teboulba Port and various fish markets along the Monastir coast. Species selection was performed randomly according to seasonal availability and market supply.

Fish species were identified based on morphological characteristics using standard taxonomic keys, with reference to regional identification guides for Mediterranean fish species (e.g., FAO species identification guides).

The number of specimens per species varied widely, ranging from 66 individuals for red mullet (*Mullus barbatus*) to a single individual for albacore (*Thunnus alalunga*) and swordfish (*Xiphias gladius*). European barracuda (*Sphyraena sphyraena*) accounted for the highest number of pooled samples (*n* = 9), whereas eleven species were represented by a single pool due to limited availability. Detailed information on all collected specimens, including habitat and feeding behavior, is provided in Table 1.

**Table 1:** Fish specimens collected with their habitat and feeding habits (Species in the Mediterranean Sea; 2015) http://www.fishbase.se/TrophicEcosysRef.php?ve_code=13

Fish species	Common name	Habitat	Feeding habits	Number of fish units	Number of pooled samples per tissue
*Mullus barbatus*	Red mullet	Demersal	Carnivorous (crustaceans, polychaetes)	66	7
*Sardina pilchardus*	European pilchard	Pelagic-neritic	Omnivorous (Phytoplankton, zooplankton)	60	7
*Sphyraena sphyraena*	European barracuda	Pelagic-neritic	Carnivorous (Fish, cephalopods)	53	9
*Trachurus mediterraneus*	Horse mackerel	Pelagic-oceanic	Carnivorous (Fish, crustaceans)	48	7
*Boops boops*	Bogue	Demersal	Omnivorous (Seaweed, crustaceans)	33	7
*Mugil cephalus*	Grey mullet	Benthopelagic	Herbivorous (Detritus, algae)	27	7
*Scorpaena notata*	Scorpionfish	Demersal	Carnivorous (Worms, fish)	27	4
*Pomatomus saltatrix*	Bluefish	Pelagic-oceanic	Carnivorous (Fish, cephalopods)	22	5
*Sparus aurata*	Gilthead seabream	Demersal	Omnivorous (Molluscs, fish)	22	6
*Echiichthys vipera*	Lesser weever	Demersal	Carnivorous (Worms, crustaceans)	22	3
*Diplodus annularis*	Annular seabream	Benthopelagic	Omnivorous (Benthic invertebrates)	22	4
*Serranus scriba*	Painted comber	Demersal	Carnivorous (Fish, crustaceans)	21	3
*Scomber scombrus*	Atlantic mackerel	Pelagic-neritic	Carnivorous (Crustaceans)	15	4
*Lithognathus mormyrus*	Striped seabream	benthopelagic	Carnivorous (Polychaetes, molluscs)	15	2
*Sarpa salpa*	Salema	Benthopelagic	Herbivorous (Seagrass, algae)	13	2
*Saurida undosquamis*	Lizardfish	Reef-associated	Carnivorous (Fish, shrimps)	12	2
*Oblada melanura*	Saddled seabream	Benthopelagic	Omnivorous (Algae, invertebrates)	12	2
*Solea solea*	Common sole	Demersal	Carnivorous (Molluscs,	10	1
*Pagellus erythrinus*	Common pandora	Benthopelagic	Invertebrates)	9	1
*Trigla lucerna*	Tub gurnard	Demersal	Carnivorous (Crustaceans, fish)	8	1
*Seriola dumerili*	Greater amberjack	Pelagic (Reef-associated)	Carnivorous (Fish, cephalopods)	8	2
*Dicentrarchus labrax*	European seabass	Demersal	Carnivorous (Small fish)	6	2
*Dentex dentex*	Common dentex	Benthopelagic	Carnivorous (Fish, molluscs	5	2
*Naucrates ductor*	Pilotfish	Reef-associated	Carnivorous (Fish, crustaceans)	5	2
*Merlangius merlangus*	Whiting	Benthopelagic	Carnivorous (Crabs, fish)	4	1
*Coryphaena hippurus*	Dolphinfish	Oceanic	Carnivorous (Fish, cephalopods)	3	1
*Uranoscopus scaber*	Stargazer	Demersal	Carnivorous (Fish, crustaceans)	3	1
*Zeus faber*	John Dory	Benthopelagic	Carnivorous (Fish, cephalopods)	2	1
*Centrolabrus exoletus*	Rock cook	Reef-associated	Carnivorous (Molluscs, fish)	2	1
*Sciaena umbra*	Brown meagre	Benthic	Carnivorous (Fish, crustaceans)	2	1
*Thunnus alalunga*	Albacore tuna	Pelagic-oceanic	Carnivorous (Fish, squid)	1	1
*Xiphias gladius*	Swordfish	Migratory	Carnivorous (Fish, molluscs)	1	1

For molecular analysis, fish samples were grouped into pools by species. Within each pool, gills, intestine, and skin–muscle tissues were aseptically dissected and processed separately. Samples corresponding to each tissue type were independently homogenized in an equal volume of TE (Tris–EDTA) buffer using an Ultra-Turrax homogenizer. Homogenates were aliquoted and stored at − 20 °C until DNA extraction.

To prevent cross-contamination, the homogenizer probe was thoroughly decontaminated between samples by washing with disinfectant solution, followed by rinsing with sterile distilled water.

### DNA extraction

Genomic DNA was extracted from 200 µL of homogenized samples, including tissue, gills, and gastric and intestinal epithelial scrapings, using the FavorPrep™ Tissue Genomic DNA Extraction Kit (Favorgen) according to the manufacturer’s instructions. To ensure efficient disruption of *T. gondii* oocysts, three freeze–thaw cycles (− 80 °C/+80 °C for 5 min each) were incorporated prior to extraction. DNA was eluted in 100 µL of elution buffer and stored at − 20 °C until further analysis.

### Real-time PCR

Detection of *T. gondii* DNA was performed using a real-time PCR (qPCR) assay targeting the 529-bp repetitive element (GenBank accession no. AF146527), as previously described (Marino et al. 2019). Each 20 µL reactional mixture contained 10 µL of 2× master mix (Thermo-Fisher Scientific, Waltham, MA), 4 µl nuclease-free water, 10 pmol of each primer (AF1: 5′-CACAGAAGGGACAGAAGT-3′ and AF2: 5′-TCGCCTTCATCTACAGTC-3′), 5 pmol of the TaqMan probe AF529 (5′−6-FAM-CTCTCCTCCAAGACGGCTGG-BHQ1-3′), 2 µL of 10× Exo IPC VIC (Applied Biosystems), 0.5 µL of 50× Exo IPC DNA (Applied Biosystems), and 40 ng of DNA template in a final volume of 20 µL for each sample. Amplification was carried out on a QuantStudio™ 7 Pro Real-Time PCR System (Applied Biosystems) with an initial incubation at 50 °C for 2 min, followed by a denaturation step at 95 °C for 10 min, and 40 cycles of amplification (95 °C for 15 s and 60 °C for 60 s). Each run included *T. gondii* genomic DNA (ATCC 50174D™, RH strain; LGC Standards) as a positive control and molecular-grade water as a negative control.

### Statistical analysis

The frequency of *T. gondii* DNA positivity across different tissues (gill, muscle/skin, intestine) was compared using Fisher’s exact test, due to some low counts (< 5) in certain tissue categories. A p-value < 0.05 was considered statistically significant. Results are presented as number of positive pools/total pools (%).

## Results

This study reports the detection of *T. gondii* DNA in marine fish from Tunisia. Out of 100 pooled samples representing a total of 559 fish analyzed, 16 pools tested positive (16%). Positive pools were detected across 13 fish species (Table 2). Gill samples were the most frequently positive, with 9 out of 16 pools (56%), including detections in *Zeus faber*,* Sparus aurata* (caged), *Sardina pilchardus*,* Sciaena umbra*,* Mugil cephalus*,* Pomatomus saltatrix*,* Mullus barbatus*, and *Lithognathus mormyrus*. Muscle/skin samples showed lower positivity, with 4 out of 16 pools (25%), detected in *Sparus aurata* (caged), *Sardina pilchardus*,* Uranoscopus scaber*, and *Sphyraena sphyraena*. Intestinal samples were the least frequently positive, with 3 out of 16 pools (19%), restricted to *Scorpaena notata*,* Serranus scriba*, and *Seriola dumerili*.

**Table 2:** Distribution of *Toxoplasma gondii* DNA in Marine Fish Species from the Tunisian Coast by Tissue Type

Species	Number of Pools	Gills Positive	Skin/Muscle Positive	Intestine Positive
*Zeus faber*	1	1/1	0/1	0/1
*Sparus aurata (cage)*	5	1/5	1/5	0/5
*Sardina pilchardus*	7	1/7	1/7	0/7
*Scorpaena notata*	2	0/2	0/2	1/2
*Sciaena umbra*	1	1/1	0/1	0/1
*Uranoscopus scaber*	1	0/1	1/1	0/1
*Mugil cephalus*	7	1/7	0/7	0/7
*Pomatomus saltatrix*	5	1/5	0/5	0/5
*Serranus scriba*	3	0/3	0/3	1/3
*Sphyraena sphyraena*	8	0/8	1/8	0/8
*Seriola dumerili*	2	0/2	0/2	1/2
*Mullus barbatus*	7	2/7	0/7	0/7
*Lithognathus mormyrus*	2	1/2	0/2	0/2

A Fisher’s exact test comparing gill versus non-gill tissues showed a significantly higher frequency of positivity in gills (*p* = 0.03; Table 2). Among species, *Mullus barbatus* showed the highest detection frequency, with 2 out of 7 pools positive (29%), while most other species had only a single positive pool. Overall, gill tissue was the most frequently positive, followed by muscle/skin and intestine.

**Fig. 1:**
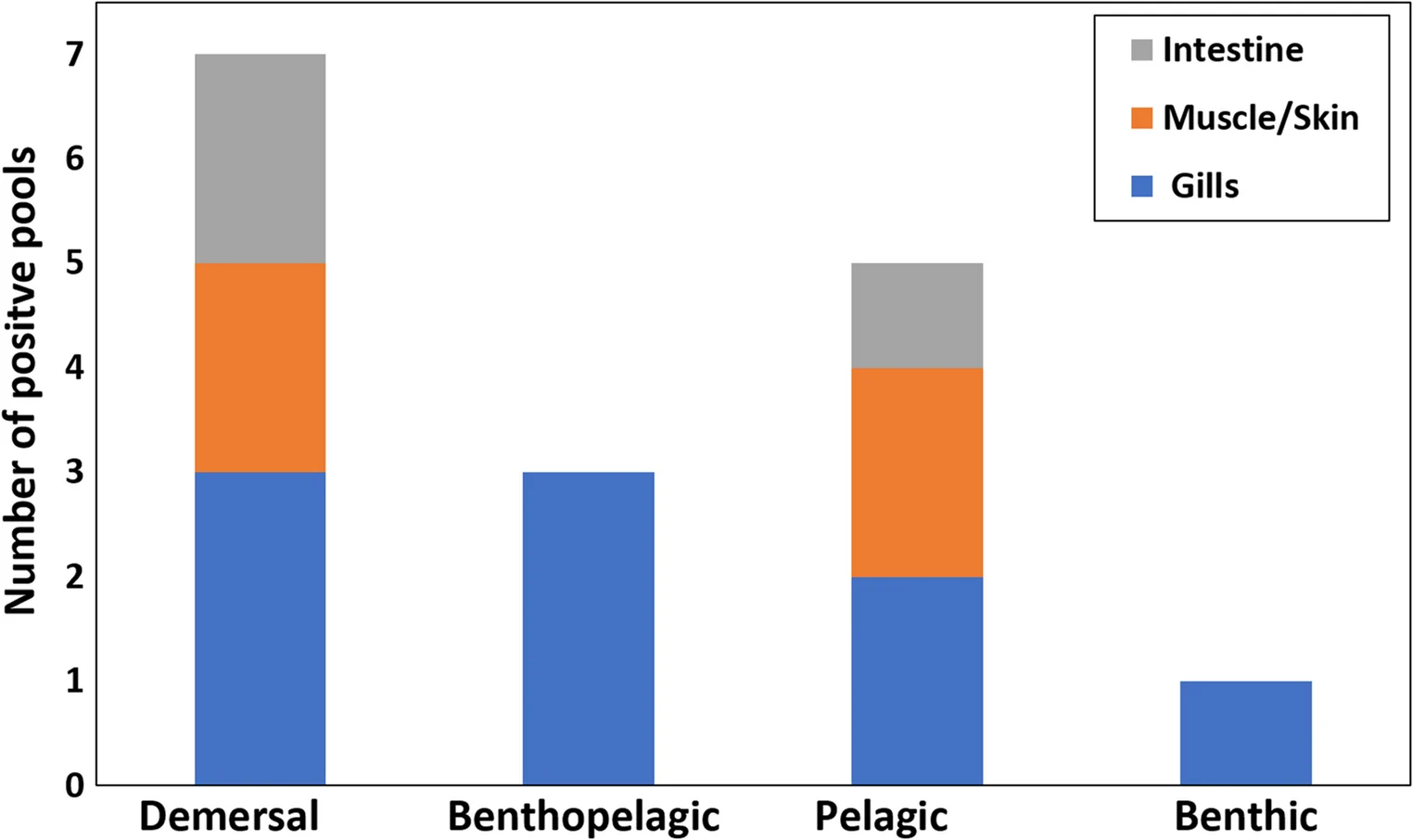
Distribution of *T. gondii* DNA positive pools by tissue type across different marine fish habitats

The distribution of *T. gondii* DNA varied slightly across different habitats (Fig. 1). Among demersal species, including *Sparus aurata*, *Scorpaena notata*, *Uranoscopus scaber*, *Serranus scriba*, and *Mullus barbatus*, 7 out of 18 pools (38.9%) tested positive, with detections distributed among gill (3 pools), muscle/skin (2 pools), and intestine tissues (2 pools). Benthopelagic species, including *Zeus faber*,* Mugil cephalus*, and *Lithognathus mormyrus*, showed 3 positive pools out of 10 (30%), with all detections occurring in gill tissues. Pelagic species, such as *Sardina pilchardus*,* Pomatomus saltatrix*,* Sphyraena sphyraena*, and *Seriola dumerili*, had 5 positive pools out of 22 (22.7%), with detections observed in gill (2 pools), muscle/skin (2 pools), and intestine tissues (1 pool). The benthic species, represented by *Sciaena umbra*, showed 1 positive pool out of 1, detected exclusively in gill tissue.

**Fig. 2:**
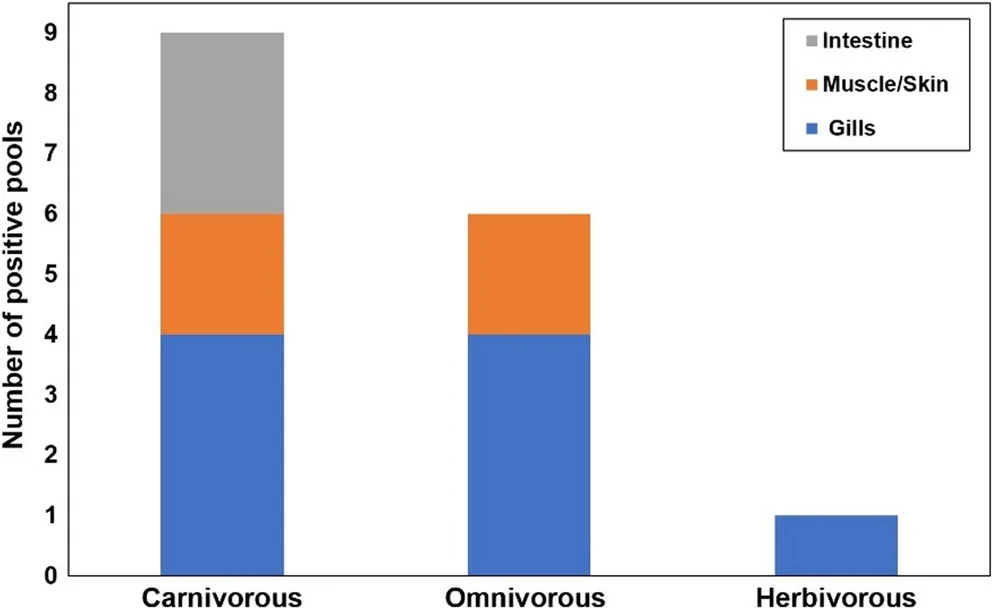
Distribution of *T. gondii* DNA positive pools by tissue type across different feeding habits

According to feeding habits (Fig. 2), carnivorous species (*Zeus faber*,* Sciaena umbra*,* Uranoscopus scaber*,* Pomatomus saltatrix*,* Serranus scriba*,* Sphyraena sphyraena*,* Seriola dumerili*,* Scorpaena notata*,* and Lithognathus mormyrus*) exhibited the highest number of positive pools, with 9 out of 25 pools (36%) testing positive, including 4 in gills, 3 in intestine and 2 in muscle/skin. Omnivorous species (*Sparus aurata*, *Mullus barbatus*,* Sardina pilchardus*) had 6 positive pools out of 19 (31.6%), with 4 positives in gills, 2 in muscle/skin, and 0 in intestine. Among herbivorous species (*Mugil cephalus*), only 1 out of 7 pools tested positive, detected in gills, while muscle/skin and intestine samples were all negative.

These results indicate that *T. gondii* contamination is present across different marine habitats and feeding behaviors, with carnivorous fish and demersal habitats showing higher detection frequencies, particularly in gill tissues.

## Discussion

The present study provides the first molecular evidence of *T. gondii* DNA in marine fish from Tunisia coast, supporting the growing recognition that marine ecosystems are increasingly impacted by land-derived contamination. The overall detection rate (16%) and the detection of the parasite DNA across 13 fish species indicate widespread environmental exposure rather than host specificity. These findings are consistent with previous studies demonstrating the persistence and stability of *T. gondii* in seawater, where the parasite can remain viable and accessible to a wide range of aquatic organisms, from invertebrates to top marine predators (Ahmadpour et al. 2022). Experimental evidence further indicates that sporulated oocysts can retain infectivity in seawater for prolonged periods, remaining viable for up to 24 months at 4 °C (Lindsay and Dubey 2009). Our results are in agreement with those reported by Marino et al. (2019), who detected *T. gondii* DNA in 12 out of 17 fish species, with 32 positive samples out of 147 (21.8%). Although the overall detection rate observed in this study (16%) is slightly lower, both studies consistently demonstrate the occurrence of *T. gondii* in marine fish.

Among *T. gondii* positive species, *Sardina pilchardus*, a small pelagic fish of major commercial importance, deserves particular attention, accounting for approximately 25% of total catches (Ben Hassine et al. 2025). The detection of *T. gondii* DNA in gills and skin of this widely consumed species, which is sometimes eaten whole or undercooked, raises potential food safety concerns. In addition, the sardine canning industry represents over 30% of total fish landings in recent years (GICA 2022), suggesting that occupational exposure may also warrant further investigation.

*Mugil cephalus*, a benthopelagic species, has been reported to harbor *T. gondii* DNA in gill tissues. Similar findings were described in Egypt by Nahnoush et al. (2022), who reported that mullet (*Mugil spp*.) accounted for 25.7% of *T. gondii*-positive samples. In that study, the parasite was detected more frequently in skin and muscle tissues than in other organs, highlighting that edible portion may contain *T. gondii* DNA. These findings suggest that mullet species may act, at least, as carriers of *T. gondii* in both freshwater and marine ecosystems, highlighting their potential role in parasite transmission to humans.

Similarly, *Mullus barbatus* was identified as another *T. gondii*-positive species, with two positive pools detected in gill tissues. This benthic fish is very common along the Tunisian coast and is harvested throughout the year. It is highly valued by Tunisian consumers and holds significant commercial importance. Comparable results were reported by Marino et al. (2019), who detected three *T. gondii*-positive gill pools in this species, confirming its potential role in parasite exposure.

The predominance of positive detections in gill tissues (9/16) strongly supports the hypothesis that fish act primarily as mechanical carriers, with gills serving as efficient filters that can trap *T. gondii* oocysts suspended in seawater. Similar findings have been reported in Nile canal fish, where the highest proportion of *T. gondii* detection occurred in gills (34.3%), followed by skin/muscle (27.1%), intestine (23.6%), and eye tissues (15%), while no brain samples tested positive, with an overall prevalence of 35% (Nahnoush et al. 2022). These consistent patterns further reinforce the role of gills as the primary site of environmental contamination and support the concept that fish are more likely to accumulate oocysts externally rather than develop systemic infection (Massie et al. 2010).

In contrast, a study on freshwater fish (*Cyprinus carpio*) conducted by Aakool and Abidali (2016) reported detection of *T. gondii* DNA primarily in enteric tissues (16/24), with limited detection in gills (2/24) and no positivity in muscle or liver samples. Conversely, Marino et al. (2019) reported higher detection frequencies in skin/muscle tissues (16/147 pooled samples), with additional detections in gills (11/147) and intestines (11/147). Such discrepancies between studies may be attributed to differences in environmental contamination levels, hydrodynamic conditions influencing oocyst distribution, species-specific ecological traits, feeding behavior, as well as methodological approaches, including sampling strategies and molecular detection techniques.

The distribution of *T. gondii*–positive samples across habitats and feeding groups in the present study is consistent with broader evidence that oocysts disperse widely, accumulate in sediments and biota, and move through food webs (VanWormer et al. 2016; Moratal et al. 2020). Although the overall detection rate was 16%, the distribution of positive pools across habitat types appeared relatively homogeneous, suggesting that contamination is not restricted to a specific ecological niche. The higher proportion of positive detections in demersal fish species (*Sparus aurata*,* Scorpaena notata*,* Uranoscopus scaber*,* Serranus scriba* and *Mullus barbatus*) supports previous findings that coastal and estuarine sediments act as important reservoirs, often harboring higher oocyst loads than the overlying water column (Li et al. 2022). Processes such as aggregation and interactions with aquatic polymers likely enhance the settling and concentration of oocysts in benthic zones, thereby increasing exposure risk for bottom-dwelling species (Shapiro et al. 2012). The detection of *T. gondii* DNA in pelagic and benthopelagic fish is also plausible, as oocysts can associate with particulates, remain suspended in the water column, and be transported over long distances by marine currents, including from highly urbanized or high–cat density watersheds (Jiang et al. 2018; Li et al. 2022). Lower detection rates in mariculture fish are consistent with more controlled conditions; however, aquaculture zones may remain vulnerable in areas affected by surface runoff and coastal pollution (Wilson et al. 2024). Overall, these findings suggest that the widespread distribution of oocysts in marine environments is likely driven by freshwater runoff, sewage discharge, and coastal contamination (Dubey 2010; Shapiro et al. 2015).

With respect to feeding behavior, the exclusive detection of *T. gondii* DNA in intestinal samples from carnivorous fish may indicate a role for trophic interactions in exposure, suggesting that ingestion of contaminated prey could be a potential route of parasite introduction in these species (De Oliveira et al. 2022; Li et al. 2022).

However, the consistent predominance of gill positivity across feeding groups in this and other studies (Nahnoush et al. 2022) supports the hypothesis that mechanical filtration from the surrounding water represents the primary route of contamination in fish, consistent with experimental evidence demonstrating efficient uptake of oocysts by aquatic organisms (Massie et al. 2010). Differences observed among trophic groups should therefore be interpreted with caution, particularly given the limited number of positive samples in some categories.

Overall, these findings support a conceptual framework in which both habitat characteristics and feeding behavior contribute to *T. gondii* exposure in marine fish, with environmental contamination playing a central role. Demersal habitats may favor sediment-mediated exposure, whereas pelagic and benthopelagic environments reflect water-column exposure dynamics. Nevertheless, mechanical retention of oocysts by gills appears to be a common and dominant mechanism across ecological groups.

These results highlight the potential role of marine fish as passive carriers of *T. gondii* oocysts and emphasize the need to consider marine transmission pathways within the broader epidemiology of toxoplasmosis. While fish are not considered true intermediate hosts, the detection of parasite DNA in their tissues raises questions about potential transmission, particularly in regions where raw or undercooked fish are consumed. Traditionally, the presence of *T. gondii* DNA in fish has been attributed to environmental contamination rather than active infection (Marino et al. 2019). However, reports of parasite DNA in internal organs such as the brain and heart, as observed in Arctic charr (Merks et al. 2024), suggest that true infection cannot be entirely excluded. Importantly, detection of DNA alone does not confirm parasite viability or infectivity; further investigations, including experimental bioassays, are required to determine whether marine fish can harbor viable and infectious stages of *T. gondii*.

## Conclusion

This study provides the first molecular evidence of *T. gondii* DNA in marine fish from the Tunisian coast, suggesting their potential role as sentinels of environmental contamination. The higher detection frequency in gill tissues indicates their suitability as a sensitive matrix for monitoring exposure to *T. gondii* oocysts in aquatic environments.

These findings support the hypothesis that marine ecosystems may act as interfaces in the land–sea transmission cycle of *T. gondii* and highlight their relevance within a One Health framework.

Future studies should focus on standardized sampling designs, larger and more representative datasets, and the integration of environmental and ecological parameters to better understand contamination dynamics.

Importantly, combining molecular detection with viability and infectivity assays will be essential to more accurately assess the public health relevance of *T. gondii* in marine food webs and its potential risk for human exposure.

## Data Availability

No datasets were generated or analysed during the current study.
